# Effect of Children’s Weight Status on Physical Activity and Sedentary Behavior during Physical Education, Recess, and After School

**DOI:** 10.3390/jcm9082651

**Published:** 2020-08-15

**Authors:** Zachary C. Pope, Charles Huang, David Stodden, Daniel J. McDonough, Zan Gao

**Affiliations:** 1 Well Living Lab, Rochester, MN 55902, USA; zachary.pope@delos.com; 2 Department of Exercise and Sport Science, Wayland Baptist University, Plainview, TX 79072, USA; huangc@wbu.edu; 3 Department of Physical Education and Athletic Training, University of South Carolina, Columbia, SC 29208, USA; stodden@mailbox.sc.edu; 4 School of Kinesiology, University of Minnesota-Twin Cities, Minneapolis, MN 55455, USA; mcdo0785@umn.edu

**Keywords:** accelerometry, body mass index, obesity, overweight, pediatrics, physical activity, physical education, sedentary behavior, segmented day

## Abstract

Children’s body mass index may affect physical activity (PA) participation. Therefore, this study examined the effect of children’s weight status on underserved elementary school children’s PA and sedentary behavior (SB) throughout the segmented day. Participants were 138 children ($\bar{X}$age = 8.14 years). Children’s height and weight were measured with subsequent classification of children as healthy weight or overweight/obese. Durations of moderate-to-vigorous PA (MVPA), light PA (LPA), and SB during physical education (PE), morning recess, lunch recess, after school, and overall were assessed via accelerometry over three days. Independent t-tests evaluated differences in children’s MVPA, LPA, and SB during each daily segment by weight status. Significantly higher MVPA was observed for children of healthy weight status versus children with overweight/obesity during morning recess, *t*(136) = 2.15, *p* = 0.03, after school, *t*(136) = 2.68, *p* < 0.01, and overall, *t*(136) = 2.65, *p* < 0.01. Interestingly, comparisons of children of healthy weight status and children with overweight/obesity’s LPA and SB during the after-school segment revealed a trend wherein children with overweight/obesity participated in slightly greater LPA/less SB than children of healthy weight status. Higher MVPA was observed among children of healthy weight versus children with overweight/obesity during most daily segments. Concerted efforts should focus on increasing MVPA among children with overweight/obesity.

## 1. Introduction

Childhood obesity continues to be a problem, particularly within developed countries like the U.S. [1]. Research has suggested overweight and obesity track from childhood through adolescence and into adulthood, with up to 77% of overweight children found to be overweight or obese as adults [2] contributing to cardiometabolic diseases (e.g., coronary heart disease, diabetes) and premature mortality [3]. Given these negative health outcomes, promotion of daily physical activity (PA) among children has become paramount [4,5,6,7]. Specifically, PA interventions may aid in the promotion of PA throughout all segments of the day (i.e., segmented day or daily segments, e.g., during recess, after school, etc.).

Body mass index (BMI) may be an important factor to consider when examining how to promote children’s PA across the segmented day as BMI has been demonstrated to influence children’s PA participation. A paucity of research has examined the potential impact that BMI has on PA participation during single daily segments [8]. For example, studies [9,10] have observed a negative relationship between BMI and after-school PA. Further, in a school-based study by Gao and colleagues [11], it was observed that children with overweight and obesity were significantly more sedentary and participated in significantly less moderate-to-vigorous PA (MVPA) than children of healthy weight status during physical education (PE). The preceding observations are congruent with other literature which has indicated a child’s BMI and/or adiposity has a negative relationship with overall daily PA participation [12,13,14]. While the cause for these observations is not fully understood, it is likely that decreased PA among children with overweight or obesity may be partially due to reduced motor skill competency [15], greater fatigability [16], and lower fitness levels [17]. Yet, few studies have examined how children’s BMI influences PA participation across multiple daily segments.

Only a handful studies [18,19,20,21] have examined PA participation during more than a single daily segment [22,23,24]. These studies, however, included notable limitations. First, most studies did not investigate BMI’s influence on PA participation during the multiple daily segments assessed. As children’s weight status may facilitate or hinder PA during certain daily segments (e.g., PE, recess, after school), investigations of BMI’s influence on children’s objectively assessed PA levels across multiple daily segments are needed. Second, some studies [18] employed pedometers as the objective PA measurement tool instead of more advanced accelerometer technology. Given the ability of accelerometry to provide information regarding time spent at different PA intensities (e.g., light PA (LPA), moderate-to-vigorous PA (MVPA)), analyses of how children’s BMI influences PA across multiple daily segments should use this technology as the PA assessment tool. This would facilitate discernment of whether children’s BMI exerts influence on PA differentially depending on PA intensity. Relatedly, as accelerometers can also provide information on sedentary behavior (SB), measurements of this outcome should also be conducted across multiple daily segments as inclusion of SB measurements would address a third notable limitation among most extant literature.

Significant positive relationships have been observed between youth SB and BMI and adiposity after controlling for confounders, such as sex, maturation, and socioeconomic status [25]. These observations are notable as SB has been found to lead to poorer health outcomes [10], with an additional concern being the tendency for PA to decline and SB to increase from childhood to adolescence [26]. Among the studies to have examined SB among children during single daily segments, the most frequently studied daily segment has been the after-school segment. A study by Atkin et al. [27] observed high levels of SB after school, with this SB comprised largely of technology-related activities, such as TV watching, video game/computer play, etc., with a sizably smaller duration of SB spent in developmentally appropriate activities, such as completing school homework. These researchers noted that while the children did engage in some PA, the mean PA duration was small relative to mean SB duration (approximately 20 vs. 68 min, respectively). Similar observations were made in a study by Colley and colleagues [10].

Resulting from the preceding literature review, investigations are needed to examine the influence of BMI on children’s PA and SB during multiple daily segments—observations which could be used to develop PA interventions during daily segments least influenced by weight status. Thus, the current study investigated the influence of children’s BMI on objectively measured SB, LPA, and MVPA during several daily segments (e.g., PE, recess, and after school). Two research questions guided the present study: (1) What are the durations of children’s SB, LPA, and MVPA during PE, recess, and after school? (2) Do children’s SB, LPA, and MVPA differ by BMI classification (i.e., weight status; healthy weight vs. overweight/obese)? Observations may provide physical educators and other health professionals information regarding which daily segments children of differing weight status might derive greatest benefit from implementation of structured PA interventions/programs.

## 2. Methods

This was a cross-sectional study in which all included data were collected at one time point. In detail, 138 first- and second-grade children (X _age_ = 8.14 years) from a Title-I public school (i.e., greater than 50% of children received free or reduce-price lunches) in Texas participated in this study in Fall 2012, with the current study part of a larger parent trial which concluded in Fall 2015. Race/ethnicity was as follows: 116 non-Hispanic White, 16 Hispanic, 4 African American, 1 Asian, and 1 undeclared. School policy mandated that children participated in structured weekly PE for 125 min and were provided a 20-min morning recess and a 30-min lunch recess. Certified PE teachers taught the PE classes. Prior to assessing children’s PA levels, school administrative support was obtained. Data were collected from 8 classes of children at the school with approximately 20 children per class. Notably, morning recess came at least 60 min after the morning PE session meaning fatigue from PE-related activities was likely not a factor in the PA and SB analyses conducted during morning recess.

The 7–10-year age range was chosen as this developmental period represents the point at which literature indicates children’s PA levels begin to decline [26,27]. Specifically, the inclusion criteria were (1) 7–10 years old; (2) children without diagnosed physical/mental disabilities; and (3) provision of written parental consent and child assent. Demographic information sheets and school records were used to verify the preceding inclusion criteria. Before data collection, Texas Tech University Institutional Review Board approval (approval #: 503217) and written parental consent and child assent were obtained, with all procedures performed with the children in accordance with the ethical standards of the Institution and/or national research committee and with the 1964 Helsinki declaration and its later amendments or comparable ethical standards [28].

Children’s PA levels were assessed with ActiGraph GT3X accelerometers (ActiGraph Corp.; Pensacola, FL, USA) which are valid and reliable for measuring children’s PA and SB in school and free-living settings [29]. Accelerometers were positioned on the right hip at the level of the superior iliac crest during all waking hours aside from time spent in activities involving water (e.g., bathing) and sleeping. Data were collected on three separate days. Fourteen hours (8:00 am–10:00 pm) were designated as children’s entire day, with 400 min (3:20 pm–10:00 pm) classified as the after-school segment, as observed in other studies of children’s segmented day [18]. Accelerometers were set to 15-s epochs with PA intensity discerned using empirically based cut-points in counts/minute (SB: 0–100; LPA: 101–2295; and MVPA: 2296 and above) [30]. Outcome variables were children’s minutes in SB, LPA, and MVPA during PE, morning recess, lunch recess, after school, and overall. The inclusion criteria for the PA data were: (1) at least 14 h of accelerometer data per day were recorded; and (2) at least three days of PA were assessed.

A Seca stadiometer (Seca, Chino, CA, USA) and Detecto digital weight scale (Detecto, Web City, MO, USA) were used to measure children’s height to the nearest half-centimeter and weight to the nearest tenth of a kilogram, respectively. The ratio of weight in kilograms and height in meters squared was used to calculate BMI, after which BMI was converted to a normative percentile based on the child’s age and sex in accordance with the Centers for Disease Control and Prevention’s (CDC) BMI-for-age growth charts [31]. Each child’s weight status was discerned from his or her BMI-for-age percentile (see Table 1). An obese classification was based on a BMI ≥ 95th %tile, and overweight was classified by a BMI ≥ 85th %tile but < 95th %tile. Children of healthy weight status had a BMI ≥ 5th %tile but < 85th %tile, children of underweight status had a BMI < 5th %tile.

Before data collection in Fall 2012, the study’s purpose and instructions on how to appropriately wear the accelerometer were explained by trained research staff. Prior to the first day of PA monitoring at the school (7:50 am–8:00 am), children were individually fit with accelerometers. If any child missed any of the three days of data collection, a make-up day was implemented. Data were downloaded from the accelerometer for each child after the child accumulated the necessary three days of wear with these data cut in accordance with the children’s schedules for daily PE, morning/lunch recess, and after school segments. Further, as the PE daily segment analyzed within the current study had more direct influence from teachers than either recess segment or the after-school segment, a brief review of the PE curriculum is advised.

During data collection, 2 certified PE teachers taught a high-quality PE curriculum focused on improving fitness through sport/game play which required the children to engage in largely continuous bouts of PA and included minimal time waiting in line. The teachers were trained by the researchers through a 30-min workshop, followed by a question-and-answer session. Later, they practiced the teaching content for one week in order to get themselves and students familiar with the content prior to the data collection session. Children engaged in sports-specific games (e.g., tag games, soccer, fitness, basketball, etc.) in four-week rotations. Throughout the data collection period, children played a game called “boccer”, combining elements of soccer and basketball, allowing children not well skilled at either sport to experience success while minimizing time children spent standing around waiting for a turn as seen in other sports (e.g., kickball). Typically, teachers reviewed the game’s rules for three minutes with a short warm-up completed thereafter. Next, balls were distributed and the boccer games began. As six baskets were positioned within the gym, six boccer games were played concurrently. During the 25-min PE class, the children’s actual engagement time in the main activity was approximately 20 min.

Accelerometer data were downloaded to ActiLife 6.12 for data sorting and processing. Data were validated for at least 12 hours of daily wear time and, again, cut to represent the daily segments of PE, morning recess, lunch recess, after school, and overall. Descriptive analyses were used to describe the characteristics of the sample, the number of children classified as healthy weight, overweight, or obese, and the number of minutes children spent in SB, LPA, and MVPA. As the main purpose of the present study was to investigate if children’s SB, LPA, and MVPA differed as a function of BMI classification (i.e., weight status; healthy weight vs. overweight/obese), we employed independent t-tests as a statistically sound and reasonable approach to compare the differences across various segments during the day. In detail, in this study, five independent t-tests were conducted to discern differences in children’s PA and SB durations during PE, morning recess, lunch recess, after school and, overall, by weight status. Significance level (α) was set at 0.05 for all statistical analyses.

## 3. Results

The final sample included 71 children of healthy weight status, 28 children classified as overweight, and 39 children with obesity. Given the relatively small group numbers of children with overweight and obesity in comparison to children of healthy weight status, the overweight and obese classifications were combined (*n* = 67) to facilitate relatively balanced data analyses. No children of underweight status were present in the sample. All children who provided written assent and had written parental consent to participate in the study wore the accelerometers for the requisite 14 h given structured follow-up procedures employed by the researchers to account for children absent on data collection days. An analysis of children’s mean time in SB, LPA, and MVPA by weight status is presented for PE, morning recess, lunch recess, and after-school segments in addition to the overall day in Table 2.

During morning recess, significantly higher MVPA was observed among children of healthy weight status compared to children with overweight/obesity, *t*(136) = 2.15, *p* = 0.03; 2.95 vs. 1.92 min, respectively, with children of healthy weight status also participating in significantly more MVPA than children with overweight/obesity during the after-school segment, *t*(136) = 2.68, *p* < 0.01; 25.38 vs. 19.81 min, respectively. Predictably, analyses of overall daily SB, LPA, and MVPA indicated significantly higher MVPA among children of healthy weight status versus children with overweight/obesity, *t*(136) = 2.65, *p* < 0.01; 47.21 vs. 39.58 min, respectively. Comparisons of children of healthy weight status and children with overweight/obesity’s mean time in SB (256.14 vs. 252.09 min, respectively) and LPA (98.78 vs. 103.16 min, respectively) after school revealed a trend toward children with overweight/obesity participating in less SB and more LPA; yet, these differences were non-significant (all *p* > 0.05). Results indicated no significant differences between weight statuses for SB, LPA, or MVPA during PE and lunch recess or for SB and LPA overall (all *p* > 0.05).

## 4. Discussion

The current study sought to investigate the influence of BMI on children’s objectively measured PA and SB levels during multiple daily segments. Nearly half (48.5%) of the children in the study were observed to be overweight or obese after classifying weight status using the CDC’s BMI-for-age growth chart [31]. This percentage is substantially higher than the percentage of children classified as overweight or obese in the U.S.—33.4% [1]. Possibly contributing to this observation is the fact the current sample was comprised of children who were underserved from a Title-I elementary school, a majority of whom came from families lower in socioeconomic standing—a factor suggested to inversely relate to youths’ BMI [32]. These observations highlight the need for continued investigation into children’s PA and SB levels throughout the segmented day and the development PA interventions for children who are underserved and/or with overweight/obesity to promote PA during all daily segments.

The study’s research questions concerned the duration of SB, LPA, and MVPA children engaged in during different daily segments and how these outcomes were influenced by BMI during each segment. Data indicated that children of healthy weight status engaged in significantly more MVPA during morning recess, after school, and overall compared to children with overweight/obesity. The BMI-related differences observed for the after-school segment are congruent with the paucity of past literature examining weight-related differences in PA and SB after school [10,21], with the weight status-related differences across the overall day also corroborated by previous literature [12,13,14,21,22]. Further, only one previous study [20] has investigated differences in children’s PA and SB during recess. Yet, this previous study did not investigate BMI-related differences during recess, rendering the observations regarding BMI-related differences in MVPA during morning recess novel and highlighting the need for additional research on the effect weight status has on PA and SB during this daily segment. Additionally, while this study’s observations of approximate one- and five-minute differences in MVPA during recess and after school, respectively, between children of healthy weight status and children with overweight/obesity are small, these figures are consistent with the literature [33]. That said, the nearly 8-min overall daily MVPA difference between children of healthy weight status and children with overweight/obesity across all daily segments evaluated is most noteworthy. Current recommendations state children should participate in MVPA on most, preferably all, days of the week for ≥ 60 min [34]. Assuming a child engages in MVPA daily, this study’s observations indicate children with overweight/obesity will participate in almost an hour less of MVPA over one week compared to their peers of healthy weight status when combining all daily segments. Nonetheless, observations still indicated that, on average, both children of healthy weight status and children with overweight/obesity fell short of the 60-min recommendation. Therefore, while continued increased concentration should be placed on increasing MVPA among children with overweight/obesity, future PA interventions should still try to facilitate increased PA among all children.

The non-significant differences between children of different weight status during PE demonstrated mixed agreement with previous literature examining weight status-related differences in PA and SB among children during this daily segment. For example, Gao and colleagues [11] observed children of healthy weight status to participate in significantly more MVPA and significantly less SB than children with overweight/obesity during PE; yet, no difference was seen between children of healthy weight status and children with overweight/obesity’s PA and SB in a PE-based study by Hannon and colleagues [35]. Observations of the current study may be partially attributable to the fitness and sport/game orientation of the class wherein teachers sought to decrease time children spent standing in line and increase activity time—engaging children in sports/games that required nearly continuous PA. Despite the PE teachers’ focus on improving fitness through sport/game play, however, it is still notable that during the 25-min PE class, children of any weight status only participated in approximately six min of MVPA—a proportion of total class time (~25%) well below recommendations stating that 50% of PE class time should be spent in MVPA [36].

While previous literature has indicated children are more active on days wherein PE is held [10,37], it is still disconcerting that children of any weight status participated in lower MVPA levels during PE [38]. Indeed, one would expect higher MVPA durations and proportions of class time spent at this PA intensity among all children given that PE represents a daily segment wherein PA should be maximally promoted. Thus, it might be prudent for schools to reimagine PA environments instead of depending exclusively upon PE. For instance, PA interventions could extend beyond PE by implementing the content promoted in PE within short classroom PA breaks and structured recess activities—perhaps employing the PE-oriented LET US Play Principles forwarded by other researchers [39]. These extensions of PE can assist non-physical education teachers in enhancing the opportunities children have to engage in PA throughout the school day. Notably, among children with overweight/obesity, a further effort may be made to promote more LPA until participating in greater MVPA is attainable as this study indicated a trend wherein children with overweight/obesity participated in greater LPA—particularly during the after-school segment—than peers of healthy weight status [40].

This study’s strengths lie in a reasonable sample size, a study sample comprised of children who are underserved, and the investigation of children’s full PA and SB spectrum via accelerometry across multiple segments of the children’s day. However, the study has limitations worth noting. First, the study was conducted in the southern region of the U.S., with many children from families of lower socioeconomic status limiting generalizability of the findings. Second, this study adopted a cross-sectional design with participants from one Title 1 school and, thus, the generalizability of the findings may be limited. Third, the present study did not require the children to document the types of SB or PAs they engaged in during either recess period or after school. Particularly, as children’s after-school measures indicated large amounts of SB, an activity log may provide a better idea of sedentary time behaviors as children may have been engaging in developmentally appropriate activities like reading/doing homework as opposed to watching TV or playing on the computer [41]. As such, future studies should examine the context of PA and SB by employing an activity log to provide more detailed analyses of the children’s PA and SB [42]. Additionally, the study only investigated children on PE days, with no comparison to non-PE days. Future studies may investigate differences across the PA and SB spectrum on PE and non-PE days in relation to weight status, as literature does suggest children’s PA patterns differ on PE and non-PE days [9,37]. Notably, the learning content was the same for physical education classes for all participants during the data collection period and thus, the confounding effects of learning content and context were minimized in this study. Yet, investigating such effects is warranted if physical education content and context is different in future studied.

The current study’s findings indicated children of healthy weight status to participate in significantly more MVPA, but not LPA or SB, than children with overweight/obesity during certain portions of the segmented day. Notably, no PA or SB differences were observed among children of differing weight status during PE—suggesting structured PA sessions may moderate the influence of weight status on PA and SB. Therefore, it might be suggested that greater concentration be placed on the development of structured PA sessions during daily segments of children’s school day which are typically devoid of structured PA (e.g., recess)—perhaps providing structured PA sessions during school-based after-school programs as well. These structured PA sessions might consider implementing PA promotion strategies aligned with public health principles as suggested in recent research [39]. Structuring environments more conducive to PA participation throughout multiple daily segments may provide an increased opportunity for all children to meet the established PA recommendation of 60 min of MVPA/day [21] which could result in weight loss or limit weight gain among children with overweight or obesity [43] and better health outcomes in adulthood.

## Figures and Tables

**Table 1:** Cutoff Points for BMI by Age and Gender *.

Age (years)	Underweight		Overweight		Obese	
	Boys	Girls	Boys	Girls	Boys	Girls
7	13.4	13.5	17.2	17.2	18.8	19.2
8	13.7	13.6	17.6	18.7	19.7	19.8
9	14	13.8	18.8	19.5	21	22.1
10	14.2	14	19.4	20	22.2	23

* Used CDC’s BMI-for-age growth charts [22].

**Table 2:** Duration of Physical Activity ^a^ by BMI during Different Daily Segments and Overall.

	Physical Education		Morning Recess		Lunch Recess		After School		Overall	
	Healthy Weight (*n* = 71)	Overwt./Obese (*n* = 67)	Healthy Weight (*n* = 71)	Overwt./Obese (*n* = 67)	Healthy Weight (*n* = 71)	Overwt./Obese (*n* = 67)	Healthy Weight (*n* = 71)	Overwt./Obese (*n* = 67)	Healthy Weight (*n* = 71)	Overwt./Obese (*n* = 67)
	*M(SD)*	*M(SD)*	*M(SD)*	*M(SD)*	*M(SD)*	*M(SD)*	*M(SD)*	*M(SD)*	*M(SD)*	*M(SD)*
SB	6.90 (3.71)	6.70 (3.46)	9.14 (4.78)	10.36 (4.33)	46.38 (11.53)	45.94 (11.82)	256.14 (55.64)	252.09 (61.12)	502.75 (89.23)	505.19 (107.49)
LPA	11.00 (2.85)	11.47 (2.96)	7.82 (2.93)	7.36 (3.19)	26.87 (8.12)	26.69 (8.82)	98.78 (34.94)	103.16 (37.38)	247.61 (56.42)	241.84 (67.29)
MVPA	6.70 (3.20)	6.15 (2.91)	2.95 (3.08) *	1.92 (2.48) *	3.57 (3.48)	3.13 (2.50)	25.38 (13.47) **	19.81 (10.65) **	47.21 (18.56) **	39.57 (14.97) **

Note: *M* = mean; *SD* = standard deviation; SB= Sedentary Behavior; LPA = light physical activity; MVPA = moderate-to-vigorous physical activity; Overwt./Obese = overweight/obese. ^a^ Physical activity in average minutes per session. * Indicates healthy weight and overweight/obese children differ at the *p* < 0.05 within segment. ** Indicates healthy weight and overweight/obese children differ at the *p* < 0.01 within segment.
